# Therapeutic effect of apatinib on overall survival is mediated by prolonged progression-free survival in advanced gastric cancer patients

**DOI:** 10.18632/oncotarget.12897

**Published:** 2016-10-25

**Authors:** Lihong Huang, Yongyue Wei, Sipeng Shen, Qianwen Shi, Jianling Bai, Jin Li, Shukui Qin, Hao Yu, Feng Chen

**Affiliations:** ^1^ Department of Biostatistics, School of Public Health, Nanjing Medical University, Nanjing, P.R. China; ^2^ Ministry of Education Key Laboratory for Modern Toxicology, School of Public Health, Nanjing Medical University, Nanjing, P.R. China; ^3^ Fudan University Shanghai Cancer Center, Shanghai, P.R. China; ^4^ People’s Liberation Army Cancer center, 81st Hospital of People’s Liberation Army, Nanjing, Jiangsu, P.R. China

**Keywords:** apatinib, gastric cancer, overall survival, progression-free survival, mediation analysis

## Abstract

Apatinib is reported to significantly improve the overall survival (OS) of patients with advanced gastric cancer who have previously failed second-line chemotherapy. However, it is not well understood whether apatinib acts by improving progression or by prolonging post-progression survival. Here, based on phase III clinical trial data, the mediating effect of apatinib on patient overall survival was systematically quantified, through progression-free survival (PFS), post-progression survival (PPS), and the disease control rate (DCR). PFS was the primary mediator of the association between apatinib treatment and OS, with an indirect-effect mean survival time ratio of 1.63 (95%CI 1.35-1.97), which mediated 93.5% of the treatment effect. The DCR was also a significant mediator among secondary efficacy endpoints, and had an indirect-effect mean survival time ratio of 1.47 (95%CI 1.20-1.79, 50.9% mediated). Both primary and other targets of the DCR had similar results. The results indicated that apatinib treatment prolongs progression-free survival rather than post-progression survival, and in turn, leads to improved overall survival. Additionally, our study highlights the value of mediation analysis in clinical trials in providing additional information to build upon traditional primary analysis.

## INTRODUCTION

Though steadily declining in prevalence over the past few decades, gastric cancer remains one of the top three most frequently diagnosed cancers and is the leading cause of cancer-related mortality, both in China and worldwide [1]. Early detection of gastric cancer has a significant impact on survival, as highlighted by the 90% five-year overall survival (OS) rate of patients whose gastric cancer is detected early. However, when diagnosed at an advanced stage, five-year OS rates range between 10-20% [2, 3]. Significant achievements have been demonstrated using first- and second-line chemotherapy for patients with advanced or metastatic gastric cancer [4]; however, after failure of second-line chemotherapy, further treatment options are limited and not widely utilized [5, 6].

Recently, we reported that apatinib, a small-molecule tyrosine kinase inhibitor that selectively inhibits vascular endothelial growth factor receptor 2, significantly improves the prognosis of patients with advanced gastric cancer who have previously failed second-line chemotherapy. Approximately one additional month of progression-free survival (PFS) and two months of prolonged OS were observed [7]. Based on these results, we proposed a new treatment option for patients involving the use of apatinib [8]. It is not clear, however, whether the OS benefit is derived from a prolonged period free of cancer progression, or from post-progression survival (PPS). This information is crucial to the timing of treatment in clinical practice, and recent studies have intensively explored this issue [9, 10]. However, heterogeneity exists across cancers [11–15].

Causal mediation analysis, a sophisticated epidemiological approach used to determine causal inference, explains the process through which the intervention (in this case, apatinib treatment) affects the outcome (overall survival) through mediators (e.g., progression-free survival) [16–18]. The total effect of the treatment comprises two parts: the indirect effect (the mediating effects) and the direct effect [19, 20]. Causal pathways from the treatment (exposure) to outcome can be uncovered in prospective epidemiological studies like clinical trials, as shown in a previous study [21].

To clarify the therapeutic benefits of apatinib, we applied mediation analysis to apatinib phase III clinical trial data (ClinicalTrials.gov identifiers NCT01512745). The mediating effect of apatinib on patients’ overall survival was systematically quantified through intermediate endpoints including PFS, PPS, and the disease control rate (DCR).

## RESULTS

### Baseline characteristics

A total of 273 patients were enrolled in the clinical trial, as described previously [22]. Briefly, five patients in the apatinib group and one in the placebo group withdrew from the study before receiving treatments. Thus, 267 patients were included in the full analysis set (FAS) population, with 176 in the apatinib group and 91 in the placebo group. Baseline characteristics are outlined in Table 1. The demographics and baseline characteristics of the two treatment groups were well balanced.

**Table 1:** Baseline demographic and clinical characteristics of full analysis set

Variable		Apatinib, *n* (%) (*N*= 176)	Placebo, *n* (%)(*N*= 91)
Age, years	Median	58	58
	Range	23-71	28-70
Sex	Male	132 (75.0)	69 (75.8)
	Female	44 (25.0)	22 (24.2)
ECOG PS	0	48 (27.3)	15 (16.5)
	1	128 (72.7)	76 (83.5)
Metastatic sites	≤2	139 (79.0)	71 (78.0)
	>2	37 (21.0)	20 (22.0)
Previous lines of chemotherapy	2	116 (65.9)	58 (63.7)
	≥3	60 (34.1)	33 (36.3)
Primary lesion	Gastric	69 (68.3)	43 (71.7)
	Gastroesophageal junction	22 (21.8)	14 (23.3)
	Unknown	10 (9.9)	3 (5.0)
Prior gastrectomy	Yes	122 (69.3)	67 (73.6)
	No	54 (30.7)	24 (26.4)
Disease stage	II	1 (0.6)	1 (1.1)
	III	10 (5.7)	5 (5.5)
	IV	162 (92.0)	83 (91.2)
	Unknown	3 (1.7)	2 (2.2)
Concomitant disease	Yes	50 (28.4)	31 (34.1)
	No	126 (71.6)	60 (65.9)

### Prognostic analysis

**Figure 1:**
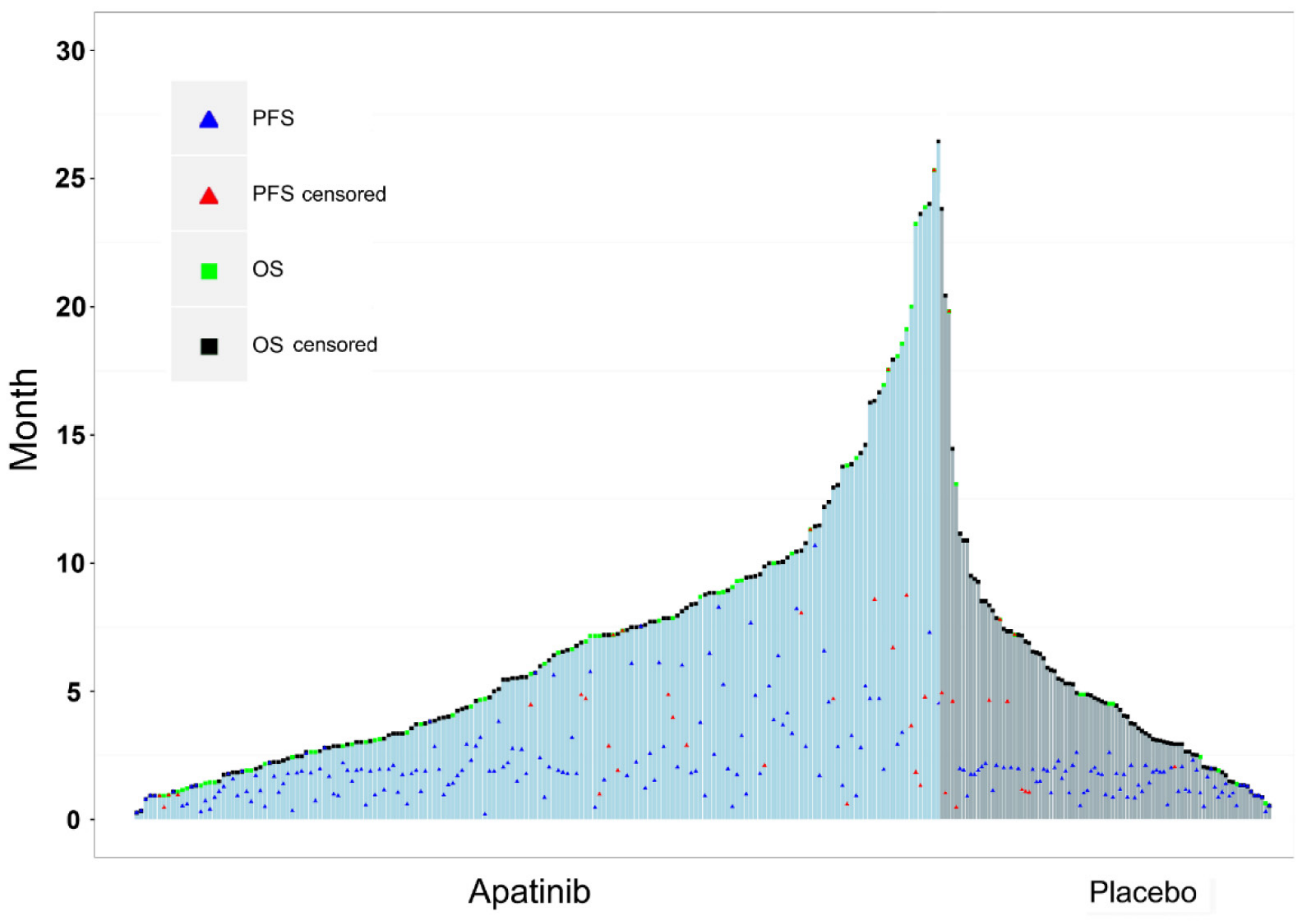
Prognosis of patients receiving apatinib or placebo treatment

Previous reports showed that median OS was significantly improved among patients in the apatinib group compared to the placebo group (6.5 months, 95% CI 4.8-7.6 *vs*. 4.7 months, 95% CI 3.6-5.4, *p* = 0.0149; hazard ratio, 0.709, 95% CI 0.537-0.937, *p* = 0.0156). Similarly, apatinib significantly prolonged the median PFS compared to the placebo (2.6 months, 95% CI 2.0-2.9 *vs*.1.8 months, 95% CI 1.4-1.9, *p* < 0.001; hazard ratio, 0.444, 95% CI 0.331-0.595, *p* < 0.001) [22]. The individual values of PFS and OS results are presented in Figure 1. Most of the PFS values were concentrated below three months for the placebo group and scattered between 0-9 months for the apatinib group.

### Mediation analysis

**Figure 2:**
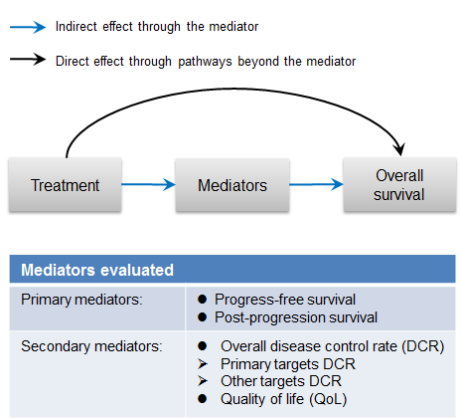
Diagram of mediation analysis

Since the patients who received apatinib treatment displayed a longer PFS and OS, we examined whether the effect of apatinib on OS was mainly mediated by the prolonged PFS, or by post-progression activity (Figure 2). Mediation analysis in the FAS population showed that PFS was a major mediator of the treatment effect on patient OS, with an indirect effect MSTR (Mean Survival Time Ratio) of 1.63 (95% CI 1.35-1.97, *p* < 0.001) that mediated 93.5% of apatinib's treatment effect (Table 2). Interestingly, after controlling for PFS, the direct effect of apatinib treatment was not significant (Table 2). In addition, after imputing the censored PFS by predicted values of the corresponding AFT model following weibull distribution, the indirect effect MSTR was 1.29, which retained statistically significant results (*p* = 0.022). On the other hand, PPS was not a significant mediator of treatment effect (*p* = 0.332). This was further supported by sensitivity analysis excluding patients with censored PFS (*p* = 0.546) (Table 2).

**Table 2:** Mediation analysis of the effect of apatinib treatment on overall survival through intermediate measures after three cycles of treatment

Mediator	Full Analysis Set						Per Protocol Set	
	N_Apatinib_	N_Placebo_	*NDE*^MSTR^(95% CI)	*NIE*^MSTR^(95% CI)	*P*	Proportion Mediated (%)	*NIE*^MSTR^ (95% CI)	*P*
**PFS**	176	91	1.03(0.81,1.31)	1.63(1.35, 1.97)	<0.001	93.50	1.72(1.34, 2.32)	<0.001
**PPS**^a^	154	79	1.30(1.11, 1.51)	1.11(0.91, 1.34)	0.332	26.67	1.16(0.97, 1.43)	0.123
**PPS**^b^	102	70	1.26(1.06, 1.49)	1.06(0.85, 1.28)	0.546	21.58	1.09(0.91, 1.32)	0.406
**DCR**								
Overall DCR	176	91	0.96(0.72, 1.28)	1.47(1.20, 1.79)	<0.001	50.91	1.54(1.25, 1.92)	<0.001
Primary target DCR	176	91	1.04(0.78, 1.39)	1.23(1.09, 1.39)	<0.001	48.91	1.30(1.11, 1.49)	<0.001
Other target DCR	176	91	1.16(0.87, 1.54)	1.09(1.00, 1.19)	0.061	46.35	1.11(1.00, 1.22)	0.045
**QoL**	176	91	1.27(0.96, 1.69)	1.00 (0.96, 1.03)	0.816	1.64	1.02(0.96, 1.07)	0.594

PFS: progression-free survival; PPS: post-progression survival; DCR: disease control rate; MSTR: mean survival time ratio. Either NDE or NIE larger than 1.00 represents a favorable survival.

^a^zero PPS excluded

^b^censored PFS excluded

**Figure 3:**
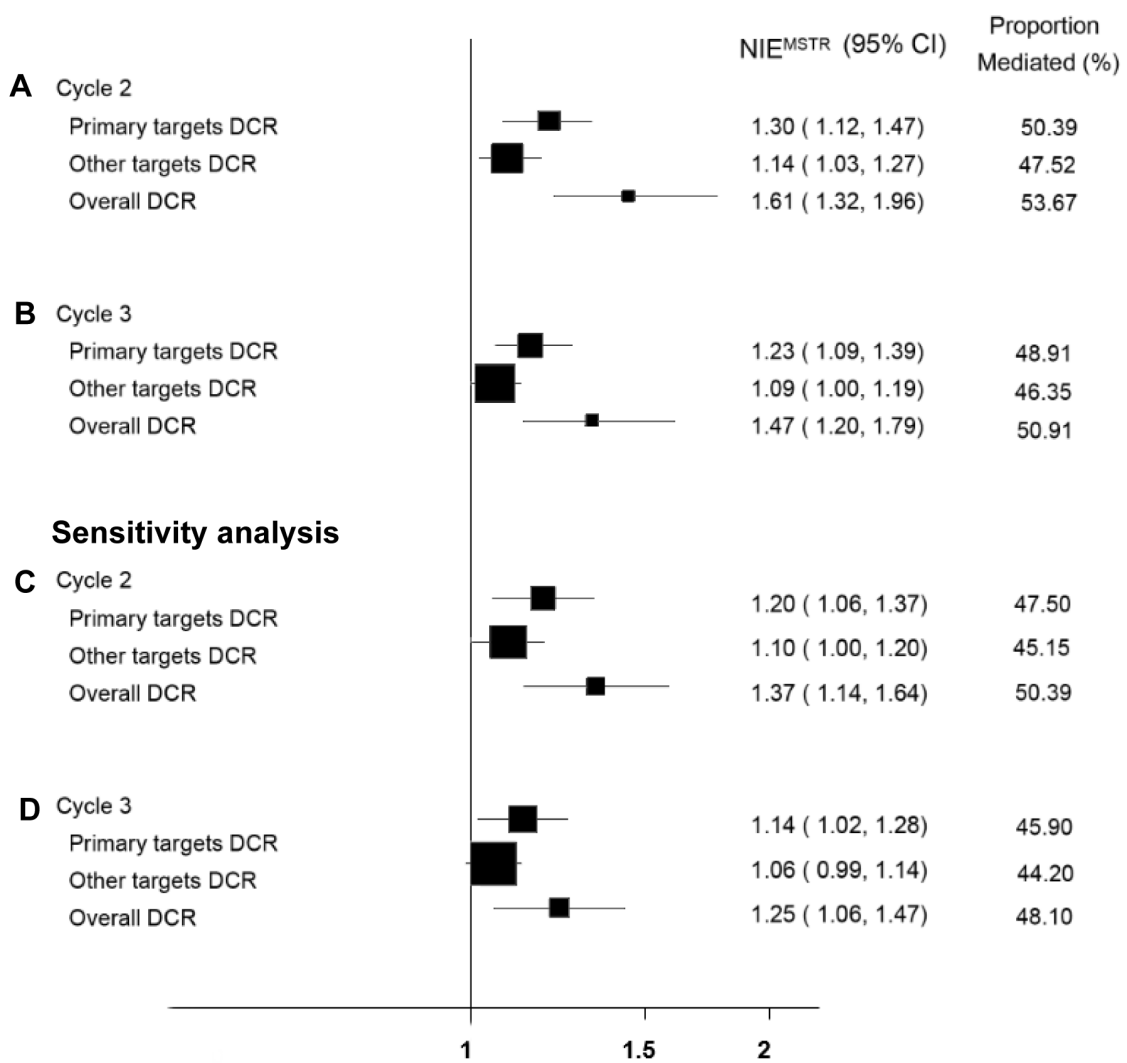
Forest plot for mediation analysis of DCR Mediation analysis was performed when patients received three **A**. or two **B**. cycles of treatment. Sensitivity analysis was performed as well by excluding patients with censored overall survival **C**., **D**.

We also evaluated two other common short-term prognostic endpoints as potential mediators—DCR (Disease Control Rate) and QoL (Quality of Life) (Figure 2). Patients in the apatinib group received an average of 2.9 cycles of medication, so we assessed DCR and QoL after three cycles of treatment for mediation analysis. About 50.91% of the apatinib treatment effect on OS was mediated by improving overall DCR (*NIE*MSTR = 1.47, 95% CI 1.20-1.79, *p* < 0.001). The primary targets’ DCR of patients receiving three cycles of apatinib treatment mediated 48.91% of the treatment's effect on patients’ OS, with statistical significance (*NIE*MSTR = 1.23, 95% CI 1.09-1.39, *p* < 0.001), while a slightly lower indirect effect was identified for other targets’ DCR (*NIE*MSTR = 1.09, 95% CI 1.00-1.19, *p =* 0.061) (Figure 3B). QoL was not a significant mediator (*NIE*MSTR = 1.00, 95% CI 0.96-1.03, *p* = 0.816). Due to small sample size and low ORR (Overall Response Rate), it was not possible to evaluate the mediating effect of ORR.

In addition, we evaluated the mediating effects of DCR when patients received two cycles of treatment. The primary targets’ DCR of patients receiving two cycles of apatinib treatment mediated 50.39% of the treatment effect on patients overall and was statistically significant (*NIE*MSTR = 1.30, 95% CI 1.12-1.47, *p* < 0.001), while a slightly lower indirect effect was identified for other targets’ DCR (*NIE*MSTR = 1.14, 95% CI 1.03-1.27, *p* = 0.013). Combining these two components, the overall DCR suggested that the indirect-effect mean survival time ratio increased to 1.61 (95% CI 1.32-1.96) (Figure 3A). However, as mentioned above, after receiving three cycles of treatment, the indirect effects were slightly decreased, as were the mediated proportions, which was likely due to more patients progressing with the disease and ceasing medication.

The sensitivity analysis, which excluded patients with censored OS, exhibited consistent results (Figure 3C, 3D). The mediated proportion (and therefore the indirect-effect mean survival time ratio) was also relatively stable when the covariates were not included in the mediation models (Table 3). Since the consistent results of FAS and PPS increase the confidence of results, sensitivity analysis on per protocol set has been performed accordingly and got similar results (Table 2).

**Table 3:** Sensitivity mediation analysis of with no covariates for the intermediate endpoints after three cycles of treatment

Mediator	N_Apatinib_	N_Placebo_	*NDE*^MSTR^(95%CI)	NIE		Proportion Mediated(%)
				*NIE*^MSTR^(95%CI)	*P*	
**Primary endpoint**						
PFS	176	91	1.04(0.79, 1.33)	1.61(1.27, 2.13)	<0.001	92.66
**Secondary endpoints**						
Overall DCR	176	91	0.99(0.74, 1.30)	1.49(1.22, 1.82)	<0.001	50.40
Primary targets DCR	176	91	1.12(0.85, 1.49)	1.20(1.08, 1.35)	0.002	47.10
Other targets DCR	176	91	1.22(0.93, 1.61)	1.09(1.00, 1.19)	0.056	44.91

PFS: progression-free survival DCR: disease control rate; MSTR: median survival time ratio. NDE or NIE larger than 1.00 represents a favorable survival

## DISCUSSION

To our knowledge, this is the first quantitative analysis of causal mediation in gastric cancer clinical trials regarding the effects of apatinib treatment on patient progression-free survival, and in turn, overall survival. Our results suggest that the overall survival benefit of apatinib treatment was mainly derived from prolonged progression-free survival, rather than post-progression survival. In addition, primary target DCR and other target DCR had similar mediation effects, while overall DCR was slightly higher, indicating that the overall DCR is evaluated by the better response targets.

The primary endpoint should be the variable capable of providing the most clinically relevant and convincing evidence directly related to the primary objective of the trial [23]. In oncology trials, OS is defined as the primary endpoint [9]. Considering that OS requires a longer follow-up time, the correlation between PFS and OS has been extensively studied in various types of cancers to evaluate the validity of PFS as a short-term surrogate. Moriwaki et al. found that the median PFS ratio was correlated with the median OS ratio in advanced biliary tract cancer [11]. Adunlin et al. indicated that PFS can be a suitable surrogate for OS in metastatic breast cancer patients [24]. Thus, PFS may be a reasonable surrogate endpoint for OS. However, in trials of HER2-targeted agents in HER2-positive metastatic breast cancer, PFS moderately correlates with OS at the individual level, suggesting that PFS does not completely substitute for OS in this setting [25]. Kasahara et al. verified that PPS has more impact on OS than PFS in recurrent small cell lung cancer patients [15]. In gastric cancer, Liu et al. reported that PFS is strongly corrected with OS based on a NICE correlation model [26]. Özer-Stillman et al. identified a strong relationship between median OS and PFS among gastrointestinal stromal tumor patients, especially in later lines of therapy based on meta-analysis [27]. However, others reached a different conclusion, questioning the validity of PFS as a surrogate endpoint for OS in gastric cancer patients [28, 29]. Despite this uncertainty, our results highlight the potential and advantage of mediation analysis in evaluating the validity and efficiency of the surrogates. Although the final decision depends on clinical evaluation, our mediation analysis supports the use of surrogates in evaluating the mediation effects beyond statistical correlation.

In addition, mediation analysis quantified the effects of several efficacy variables in clinical trials. For this study, among secondary efficacy endpoints, DCR (overall) was the one and only mediator. QoL was not an effective mediator, indicating that apatinib may not prolong OS by improving the quality of life. The primary endpoint of a confirmatory trial nearly always rests on earlier clinical work carried out in a series of exploratory trials with several different efficacy endpoints [23]. Although the final choice of the primary endpoint depends on clinical evaluation, the mediation analysis results are objective and could be used as supporting analysis in exploratory trials to avoid subjective bias from clinicians.

There are several limitations to our study. First, the validity of our results relies on the assumption that there were no unmeasured confounding variables. To have valid estimates of natural direct and indirect effects, we assumed that there was no unmeasured confounding effect on (1) treatment-OS, (2) mediator-OS, or (3) treatment-mediator, and that there were (4) no mediator-OS confounders effected by treatment [30]. The clinical trial included patients according to the inclusion and exclusion criteria, performed randomization, and was well controlled throughout the trial. Therefore, potential confounders have been controlled. However, the findings were obtained from only one clinical trial; thus, more well studied trials are needed to verify the advantage of the mediation analysis. In addition, the observed strong correlation between PFS and OS in the current study probably be specific for patients have had at least two lines of chemotherapy fail before participating in the study. Thus, the finding should be interpreted with caution among patients receiving first- or second-line treatments.

## MATERIALS AND METHODS

### Study population

The study population was described previously [22]. Briefly, study randomization was stratified according to the number of metastatic sites (more than two sites *versus* up to two sites). The 273 patients from 32 sites in China, ages 18-70, were histologically confirmed as having advanced or metastatic gastric or gastroesophageal junction adenocarcinoma. Patients were randomized by 2:1 to a study group that received apatinib (850 mg once daily) (N = 181) or a placebo (N = 92). All participants had at least two lines of chemotherapy fail before participating in the study. Additional enrollment criteria were as follows: at least one measurable lesion as defined by the Response Evaluation Criteria in Solid Tumors (RECIST, version 1.1), Eastern Cooperative Oncology Group (ECOG) performance status of 0 or 1, and acceptable hematologic, hepatic, and renal function. All patients gave their written informed consent, and approval was obtained from the relevant ethical committees.

There were two primary endpoints: OS and PFS. The time interval before progression or death was PFS. OS was defined as the duration from the time of random assignment to the time of death. Secondary efficacy endpoints included the objective response rate (ORR; including the rate of complete response plus partial response), DCR (including complete response, partial response, and stable disease), and quality of life (QoL; determined using the validated European Organization for Research and Treatment of Cancer Quality of Life Questionnaire Core 30, EORTC QLQ-C30).

A treatment cycle was defined as 28 days (four weeks). Tumor assessments (RECIST) were performed at baseline, after cycles two and three, and every eight weeks thereafter until disease progression was detected. Radiological assessment for disease progression was determined by five independent radiologists from different hospitals. QoL was assessed at baseline, after cycles two and three, and every eight weeks thereafter until disease progression was detected, death occurred, or consent was withdrawn.

### Exposure, mediators, outcomes, and covariates

In this clinical trial, apatinib or placebo treatment was considered to be the exposure variable while the outcome was OS. The PFS was considered as the primary mediator in the mediation analysis (Figure 2), as it was the surrogate endpoint for OS in some oncology clinical trials [13, 31]. PPS was also evaluated as a potential mediator (Figure 2). To quantify the indirect effects of secondary efficacy endpoints, DCR, and QoL were the analyzed as well (Figure 2).

We considered age, ECOG status (0 *vs*. 1), previous chemotherapy (three or more *vs.* two chemotherapy lines), and number of metastatic sites (more than two *vs*. two or fewer sites) as covariates, in accordance with the multiple Cox model analysis in the original statistical analysis report [7].

### Statistical analysis

There were 3 types of mediators evaluated in this study: continuous (QoL), binary (DCR and ORR), and time-to-event (PFS and PPS). Baseline measurements including age, ECOG status, previous chemotherapy, and number of metastatic sites were considered as covariates (c) in the following models.

#### Mediation analysis for continuous mediators

We used the two-step regression method proposed by VanderWeele to estimate the natural direct effect (*NDE*) and natural indirect effects (*NIE*) of apatinib [32, 33]. The mediator model was defined as

$$M=\beta_{0}+\beta_{1}\cdot treatment+\beta_{2}\cdot c+e$$

Assuming OS followed the accelerated failure time (AFT) model, the outcome model was defined as:

$$\log\left(\text{OS}\right)=\theta_{0}+\theta_{1}\cdot treatment+\theta_{2}\cdot M+\theta_{3}c+v\varepsilon$$

where *c* represents covariates, and *ε* follows the extreme value distribution and *v* is a shape parameter with *v* = 1 if an exponential distribution is assumed or other values if a Weibull distribution is assumed. The *NDE* and *NIE* in the scale of mean survival time ratio (MSTR) are defined as:

$$\begin{array}{l} NDE^{MSTR}=\exp\left(\theta_{1}\right)\\ NIE^{MSTR}=\exp\left(\beta_{1}\cdot \theta_{2}\right) \end{array}$$

#### Mediation analysis for binary mediator

The mediator model was re-defined as:

$$\text{logit}\left\{P\left(M=1|A,C\right)\right\}=\beta_{0}+\beta_{1}\cdot treatment+\beta_{2}c$$

The NDE and NIE were estimated by:

$$NDE^{MSTR}=\frac{\exp(\theta_{1})\{1+\exp(\theta_{2}+\beta_{0}+\beta_{2}c)\}}{\left\{1+\exp(\theta_{2}+\beta_{0}+\beta_{2}c)\right\}}=\exp(\theta_{1})$$

$$NIE^{MSTR}=\frac{\left\{1+\exp(\beta_{0}+\beta_{2}c)\}\{1+\exp(\theta_{2}+\beta_{0}+\beta_{1}+\beta_{2}c)\right\}}{\left\{1+\exp(\beta_{0}+\beta_{1}+\beta_{2}c)\}\{1+\exp(\theta_{2}+\beta_{0}+\beta_{2}c)\right\}}$$

#### Mediation analysis for time-to-event mediator

Assuming both mediator and outcome (OS) followed the accelerated failure time (AFT) models, the mediator and outcome models were re-defined as:

$$\log\left(M\right)=\beta_{0}+\beta_{1}\cdot treatment+\beta_{2}c+v\varepsilon$$

$$\log\left(\text{OS}\right)=\theta_{0}+\theta_{1}\cdot treatment+\theta_{2}\cdot \log\left(M\right)+\theta_{3}c+v\varepsilon$$

Here, the *NDE*MSTR and *NIE*MSTR were estimated by:

$$\begin{array}{l} NDE^{MSTR}=\exp\left(\theta_{1}\right)\\ NIE^{MSTR}=\exp\left(\beta_{1}\cdot \theta_{2}\right) \end{array}$$

In addition, the proportion of the treatment effect mediated by each variable was calculated by the natural indirect effect divided by the total effect [34]. This metric evaluates the degree to which the treatment's success was influenced by mediators.

In sensitivity analyses, we excluded the pre-defined covariates to evaluate their impact on the results of the clinical trials. We also restricted the analysis to observations with non-censored OS to better control for confounding by censoring.

Statistical analyses were performed in the full analysis set and conducted using SAS macro software released by Valeri and VanderWeele [35] and R software Version 3.2.3 (The R Foundation for Statistical Computing).

## Abbreviations

DCR: disease control rate
ECOG PS: Eastern Cooperative Oncology Group Performance status
MSTR: mean survival time ratio
NDE^MSTR^: mean survival time ratio scale of natural direct-effect
NIE^MSTR^: mean survival time ratio scale of natural indirect-effect
ORR: overall response rate
PFS: progression-free survival
PPS: survival post-progression
QoL: quality of life
